# Immunomodulatory Effects of Nintedanib on Human Blood Monocytes/Macrophages from Patients with Idiopathic Pulmonary Fibrosis

**DOI:** 10.3390/biom16020319

**Published:** 2026-02-18

**Authors:** Maria Talmon, Arianna Mares, Hari Baskar Balasubramanian, Chiara Mocchetti, Lara Camillo, Piero Balbo, Luigia Grazia Fresu, Filippo Patrucco

**Affiliations:** 1 Department of Pharmaceutical Sciences, University of Piemonte Orientale, Via Bovio 6, 28100 Novara, Italy; maria.talmon@med.uniupo.it; 2 Department of Health Sciences, School of Medicine, University of Piemonte Orientale, Via Solaroli, 17, 28100 Novara, Italy20041240@studenti.uniupo.it (H.B.B.); lara.camillo@uniupo.it (L.C.); luigia.fresu@med.uniupo.it (L.G.F.); 3 Division of Respiratory Diseases, Maggiore della Carità University Hospital, C.so Mazzini 18, 28100 Novara, Italy

**Keywords:** IPF, idiopathic pulmonary fibrosis, nintedanib, monocyte, macrophage, oxidative stress

## Abstract

Background: Nintedanib (NTD) is an inhibitor of several tyrosine kinases whose role in the pathogenesis of idiopathic pulmonary fibrosis (IPF) is well recognized. Therefore, NTD was approved for the management of IPF about ten years ago. NTD has been demonstrated to have immunomodulatory effects in vitro. We now evaluated the effects of NTD on monocyte/macrophage phenotype isolated from IPF patients treated with NTD. Methods: Monocytes were isolated from IPF patients naïve for treatments and used as such or differentiated into M1- and M2-like macrophages. The cellular phenotype (characterized by the expression pro- and anti-fibrotic surface markers) and responsiveness (characterized by oxidative stress and cytokine expression/release) were evaluated, at T0 (before treatment starts) and after 6 months of treatment with a 150 mg capsule of NTD twice a day (T1). Results: Following differentiation, both M1 and M2 macrophage populations, derived from monocytes isolated from patients treated with NTD, present a higher percentage of cells positive for anti-fibrotic CD80/CD86 and expressing less profibrotic CD206/CD163. Importantly, gene expression and release of the pro-fibrotic factor TGF-β were significantly decreased at T1. Conclusions: These results show that although it does not have a direct effect on monocyte phenotype/responsiveness, NTD in vivo appears to prime monocytes to differentiate preferentially towards an anti-fibrotic macrophage phenotype, suggesting that it has an immunomodulatory effect on macrophage polarization. This data leads us to hypothesize that NTD could also induce this change in vivo, thus contributing to the improvement of the patient’s fibrotic state.

## 1. Introduction

Idiopathic Pulmonary Fibrosis (IPF) is a fibrosing, chronic and progressive interstitial lung disease [1], that is characterized by the presence of a radiological and histopathological pattern of usual interstitial pneumonia (UIP), in the absence of alternative etiology for this pattern [2]. A typical UIP pattern on high-resolution computed tomography (UIP-CT) is defined by the presence of honeycombing and reticular opacities, with or without traction bronchiectasis, with a predominance of the subpleural and basal area of both lungs [1] otherwise, the UIP-CT’s probable pattern is characterized by subpleural, basal-predominant reticular abnormalities with peripheral traction bronchiectasis or bronchiolectasis, without honeycombing [3]. IPF causes a reduction in lung volume that can be measured by the reduced total lung capacity (TLC), therefore identifying a functional restrictive pattern [4]; forced vital capacity (FVC) is accepted as a surrogate of disease severity and progression, and for this reason, it has been largely used as a standard spirometric measure of pulmonary function in IPF patients [5]. Finally, the diffusing capacity of the lungs for carbon monoxide (DLCO) measures the ability of the lungs to transfer gas from inspired air to the bloodstream [6]; in IPF patients, DLCO is almost always reduced, even in cases of normal TLC or FVC [7].

The anti-fibrotic drug nintedanib (NTD) has been approved for the management of IPF in 2014 [8,9] for its ability to reduce the annual decrease in FVC and acute exacerbations in patients [10,11,12]. NTD is a tyrosine kinase inhibitor that exerts a triple action mode, namely by blocking the ATP-binding sites within the kinase domains of the Vascular Endothelial Growth Factor (VEGF), of the basic Fibroblast Growth Factor (bFGF) and of the Platelet-Derived Growth Factor (PDGF) receptors [13], thus inhibiting the PDGF-, VEGF- or bFGF-dependent proliferation of fibroblasts [14]. In addition, several studies have shown that NTD prevents the development of inflammatory processes during fibrosis in murine models [15,16,17]. In fact, NTD markedly prevents the expression of major pro-inflammatory and pro-fibrotic cytokines, such as tumor necrosis factor-α (TNF-α), interleukin-(IL-)1β, IL-6 or chemokine ligand (CCL) 2, in fibrotic livers, lungs and kidneys. Moreover, it has been demonstrated that NTD prevents in vitro pro-fibrotic macrophage polarization [18] and inhibits the alternative activation of monocytes, reducing CD163 and CD206 expression in macrophages differentiated from pre-treated monocytes [18,19].

The polarization properties of NTD are of great interest as a large body of evidence supports the concept that an excess of M2 macrophages can promote fibrosis [19,20]. As is known, the common classification divides the macrophages into two phenotypes: (i) classically activated M1 macrophages that result from the TNFα and/or INFγ stimulus; and (ii) alternatively activated M2 macrophages that arise from an IL-4-, IL-10-, IL-13- and TGFβ-enriched environment [21,22]. Macrophage polarization is a dynamic process, thus allowing macrophages to switch from one phenotype to the other to adapt to the external milieu [23,24]. In an IPF context, the role of macrophages is well recognized, but the mechanism is still not completely understood [25]; activated M1 macrophages have a role at the early stage of IPF, releasing elevated amounts of pro-inflammatory cytokines, therefore inducing chronic inflammation and tissue damage [26]. Activated M2 macrophages, which express specific surface markers such as CD206, CD163, YM-1, and Arg-1, secrete anti-inflammatory and pro-fibrotic cytokines, mainly IL-10, PDGF, and TGF-β, then begin to prevail over the M1 population, favoring the proliferation of fibroblasts and uncontrolled repair mechanisms that lead to progressive fibrosis, associated with reduced lung function in the advanced fibrosis stage of IPF, resulting in severe fibrosis [26]. Therefore, making the environment more favorable for the classically activated macrophages could help to slow down the progression of fibrosis in IPF patients by reducing the exacerbation of disease symptoms. Several pre-clinical studies in vivo have demonstrated that the inhibition of M2 macrophages hinders IPF development [27,28], paving the way for a new pharmacological strategy for IPF that targets M2 macrophage polarization. Due to the difficulties in identifying new molecules with a specific mechanism able to balance the polarization of the macrophages between the M1 and M2 phenotype, the purpose of this study is to evaluate whether NTD, besides its tyrosine kinase inhibitory action, is endowed with immunomodulatory properties too, by analyzing the phenotype and responsiveness of peripheral monocytes and monocyte-derived macrophages from patients affected by IPF, after long-term NTD treatment. Therefore, the objective of the research was to evaluate the effects of NTD on the difference in responsiveness and phenotype of monocytes/macrophages among the drug-naïve patients with IPF from baseline to 6-month follow up. Our results demonstrate the ability of NTD to prime circulating monocytes toward an anti-fibrotic macrophage phenotype.

## 2. Materials and Methods

### 2.1. Patient Enrolment and Treatment

We enrolled 20 patients attending the Division of Respiratory Diseases Medical Department of the AOU Maggiore della Carità Hospital (Novara, Italy). All subjects gave their informed consent for inclusion before participating in the study. The study was conducted in accordance with the Declaration of Helsinki, and the protocol was approved by the Ethics Committee of Azienda Ospedaliera Maggiore della Carità, Novara (Protocollo 264/CE). Moreover, the study was conducted in accordance with the Strengthening the Reporting of Observational studies in Epidemiology (STROBE) statement for observational studies [29]. IPF diagnosis was made according to international guidelines and all patients were discussed by the multidisciplinary team of our institute [3]. The inclusion criteria established definite IPF following international guidelines [3], in patients naïve for any anti-fibrotic treatment and eligible for IPF treatment with NTD. Patients must have a measured forced vital capacity (FVC) ≥ 50% and diffusion lung for CO (DLCO) ≥ 30%. We excluded from the study patients without a definite diagnosis of IPF, those not eligible for NTD, or patients already in treatment with pirfenidone. During the enrolling phase (T0), the following data have been recorded: demographics (age, gender, and body mass index), comorbidities (i.e., cardiological, respiratory, oncologic, endocrinological, nephrological), pulmonary function (FVC, forced expiratory volume in one second—FEV1, and total lung capacity—TLC, DLCO), GAP (gender, age, and lung physiology) index [30], and detailed cell blood count (with white and red blood cells, neutrophils, lymphocytes, monocytes, and eosinophils).

Within a month from IPF diagnosis, NTD 150 mg capsules were administered orally twice daily.

The baseline characteristics of patients are shown in Table 1. At T0, most of the patients were functionally mildly restricted, with a mean TLC of 63.3% (±18.5%), FVC of 70.8% (±13.1%) and moderately reduced DLCO (52.5 ± 12.7%). The majority of patients were ex-smokers, with a normal BMI. The UIP-CT probable pattern was the most represented (55%). The mean peripheral count of monocytes was 0.58 ± 0.2 cells/µL.

### 2.2. Monocyte Isolation and Differentiation

Blood samples have been collected by venipuncture before starting the treatment with NTD (T0) and after six months from the first dose (T1). Monocytes were isolated by the standard technique of dextran sedimentation and Histopaque (density = 1.077 g cm^−3^, Sigma-Aldrich, St. Louis, MO, USA) gradient centrifugation (400× *g*, 30 min, RT) and recovered by thin suction at the interface, as described previously [31]. Purified monocyte populations were obtained by adhesion (90 min, 37 °C, 5% CO_2_) in serum-free RPMI 1640 medium (Sigma-Aldrich, St. Louis, MO, USA) supplemented with 2 mM glutamine and 1% of penicillin–streptomycin (Life Technologies, Milan; Italy). Monocytes were partly used for the analysis described below and partly differentiated into M1 and M2 monocyte-derived macrophages (MDM). To differentiate monocytes into M1-like macrophages, cells have been cultured in 10% FBS-enriched medium with hrGM-CSF (50 ngmL^−1^) for 5 days, and then INFγ (20 ngmL^−1^) and LPS (50 ngmL^−1^) were added for an additional 24 h. To obtain M2-like macrophages, monocytes were cultured in 10% FBS-enriched medium added by hrM-CSF (50 ngmL^−1^) for 5 days, and then hrIL4, hrIL13 and hrIL10 (20 ngmL^−1^, all cytokines Immunotools, Friesoythe, Germany) were added for an additional 24 h. Cell phenotype characterization was evaluated by the expression of specific surface proteins CD86/CD80 and CD206/CD163, as pro- and anti-inflammatory markers, respectively.

### 2.3. Superoxide Anion (O_2_^−^) Production

Cells (1 × 10^6^ cells/plate) were plated and O_2_^−^ production was evaluated by the superoxide dismutase (SOD)-sensitive cytochrome C reduction assay and expressed as nmoles reduced cytochrome C/10^6^ cells/30 min, using an extinction coefficient of 21.1 mM.

Moreover, we evaluated the percentage of cells producing oxygen and nitrogen reactive species by FACS analysis, using the Cellular ROS/Superoxide Detection Assay Kit (AbCam, Cambridge, UK) according to the manufacturer’s instructions. The results were analyzed by an Attune NxT Flow Cytometer (Life Technologies, Milan, Italy).

### 2.4. Flow Cytometry Analysis

Measurement of surface marker expression was performed by flow cytometry multi-parametric analysis (Attune NxT Flow Cytometer, Life Technologies) using different antibody panels as detailed below. The possible fluorochrome overlap has been avoided by compensation. Monocytes have been stained using APC anti-hrCD14 (host: mouse, clone: 61D3) and FITC anti-hrCD16 (host: mouse, clone: CB16) antibodies (all Invitrogen, Carlsbad, CA, USA). The cell population was first defined using forward scatter (FSC) and side scatter (SSC) to find viable cells and exclude debris (Supplementary Figure S1A). In this population, the co-expression of CD14 and CD16 markers was analyzed on a dot plot (Supplementary Figure S1B). For MDM, the following antibody panels have been used: (i) APC anti-hrCD14, FITC anti-hrCD80 (host: mouse, clone: 2D10.4) and PE anti-hrCD86 (host: mouse, clone: IT2.2); and (ii) APC anti-hrCD14, PE anti-hrCD163 (host: mouse, clone: GHI/61), and PerCp anti-hrCD206 (host: mouse, clone: 19.2, all Invitrogen, Carlsbad, CA, USA). First, we selected CD14+ cells (Supplementary Figure S2A), and in this population, we evaluated both the single and double positive cells for CD80 and CD86 (Supplementary Figure S2B) and CD163 and CD206 (Supplementary Figure S2C). Gates were defined using the unstained control. Data were therefore expressed as the number of positive cells over the CD14+ population number.

### 2.5. Quantitative Real-Time PCR

The expression of TGFβ, IL-1β, IL-6, IL-4, IL-10 and CCL22 was evaluated by qRT-PCR on monocytes and MDM at T0 and T1. Total RNA was isolated by Trizol Reagent. 1 µg of RNA was retrotranscribed by a high-capacity SensiFAST™ cDNA Synthesis Kit (Bioline, Milan, Italy) according to the manufacturer’s instructions. A two-step cycling real-time PCR was carried out in a volume of 10 μL per well in a 96-well optical reaction plate (Biorad, Milan, Italy) containing SensiFast No-ROX kit (Bioline, Milan, Italy) 1x, forward and reverse primer 400 nM, and 1 μL of cDNA template. GAPDH was used as internal control. The sequences of primers are as follows. TGFβ for 5′-TGATGTCACCGGAGTTGTGC-3′, rev: 5′-GTGAACCCGTTGATGTCCACT-3′; IL-1β for 5′-ACAGATGAAGTGCTCCTTCCA-3′, rev: 5′-GTCGGAGATTCGTAGCTGGAT-3′; IL-6 for 5′-GGAGACTTGCCTGGTGAAAA-3′, rev: 5′-GTCAGGGGTGGTTATTGCAT-3′; IL-4 for 5′-CCGTAACAGACATCTTTGCTGCC-3′, rev: 5′-GAGTGTCCTTCTCATGGTGGCT-3′; IL-10 for 5′-CATCGATTTCTTCCCTGTGAA-3′, rev: 5′-TCTTGGAGCTTATTAAAGGCATTC-3′; CCL22 for 5′-CACTCCTGGTTGTCCTCGTC-3′, rev: 5′-CAGCAGACGCTGTCTTCC-3′; and GAPDH for 5′-AACGTGTCAGTGGTGGACCTG-3′, rev: 5′-AGTGGGTGTCGCTGTTGAAGT-3′.

### 2.6. ELISA

The culture medium of monocytes/macrophages at T0 and T1 were collected. Secreted cytokine levels were evaluated by the ELISA Kit (Invitrogen, Carlsbad, CA, USA) following the manufacturer’s instructions. The reference ranges of the kits are as follows. hTGFβ: 31–2000 pg/mL; hIL-6: 7.8–500 pg/mL; hIL-1β: 7.8–500 pg/mL; hIL-10: 20.5–1976 pg/mL; and hIL-4: 7.8–500 pg/mL.

### 2.7. Statistical Analysis

Data and statistical analysis comply with the recommendations on experimental design and analysis in pharmacology [32]. Data are presented as box-and-whisker plots showing the median (center line), interquartile range (box) and minimum–maximum values (whiskers) of ‘*n*’ independent experiments. Data were analyzed by Student’s t test or respective non-parametric testing as reported in figure captions based on the analysis of data distribution and homogeneity of variance. A value of *p* < 0.05 was considered statistically significant.

## 3. Results

### 3.1. Changes in Pathological Features of IPF Patients After 6 Months of NTD Treatment

Enrolled patients were diagnosed with IPF according to international guidelines [3]. At T1, all patients were in treatment with NTD (150 mg twice daily) for six months: FVC, TLC and DLCO were meanly decreased by 2.6%, 6.0% and 10.2%, respectively (Supplementary Table S1).

Despite the limited number of patients enrolled, they were stratified by UIP-CT pattern, FVC, TLC, and DLCO to characterize basal monocytes and MDM phenotypes and respiratory burst activity. We were well aware of the low statistical power of the results we would obtain, but they could still provide us with an indication of a trend of action.

Monocytes are classified as classical (CD14++CD16−), non-classical (CD14+CD16+), and intermediate (CD14++CD16+), having different functional roles and expression in various inflammatory diseases [33,34,35,36]. Patients with a definite UIP-CT pattern showed a significant decrease in favor of the intermediate ones (CD14+CD16+) compared to patients with a probable UIP-CT (Supplementary Table S2A). M1 and M2 populations did not differ between groups, and no associations were found with FVC or TLC. Basal oxidative stress was higher in monocytes and M2-like MDMs from patients with definite UIP-CT (Supplementary Table S2B). Stratification by DLCO showed no differences in monocyte subset distribution, but patients with DLCO < 40% exhibited reduced CD80+CD86+ and slightly increased CD163+CD206+ expression in M2-like MDMs (Supplementary Table S3A), along with increased basal superoxide production in monocytes and M2-like MDMs (Supplementary Table S3B). No significant differences were observed when stratifying by monocyte count or FVC (Supplementary Tables S4 and S5).

### 3.2. NTD Drives Macrophage Polarization Towards a Pro-Inflammatory Phenotype

After 6 months from the first dose (T1), we then analyzed the effect of the treatment with NTD on monocytes and MDM phenotype.

**Figure 1:**
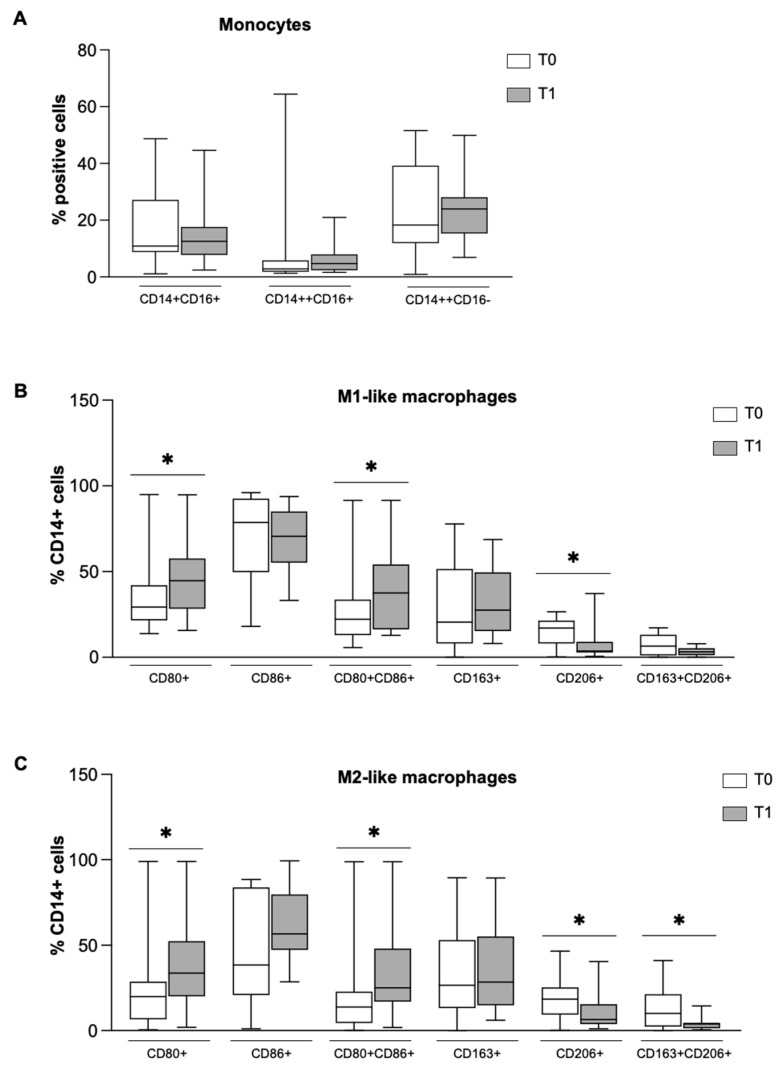
Effect of NTD on the phenotype of monocytes (**A**), M1-like macrophages (**B**) and M2-like macrophages (**C**) at T0 (white bar) and T1 (gray bar). (**A**) FACS analysis of monocyte populations: CD14+CD16+, CD14++CD16+ and CD14++CD16−. (**B**) FACS analysis of M1-like macrophage population. (**C**) FACS analysis of M2-like macrophage population. Data were analyzed by the Wilcoxon–Mann–Whitney test and presented as box-and-whisker plots showing the median (center line), interquartile range (box) and minimum–maximum values (whiskers) of the percentage of positive cells on the CD14+ population from 16 distinct patients. Statistical significance: * *p* < 0.05 versus T0.

To evaluate if the long-term treatment with NTD could exert immunomodulatory effects by imprinting monocytes to differentiate preferentially versus M1- or M2-like macrophages, we performed cytofluorimetric analysis of surface markers expressed by monocytes and derived macrophages in the naïve state (T0) and after 6 months of drug treatment (T1). Although the results did not reveal significant differences between monocyte subsets (Figure 1A), thus demonstrating that NTD did not induce an apparent effect on monocytes, the analysis showed the ability of NTD to drive macrophage polarization. Indeed, despite the in vitro experimental conditions of an environment favorable to differentiation versus M1-like macrophages (Figure 1B) or M2-like macrophages (Figure 1C), the MDM from patients under treatment with NTD for 6 months presented a significant increase in cells positive for the M1 markers CD80 and CD86 and a significant reduction for the pro-fibrotic M2 markers CD206 and CD163 in both conditions.

**Figure 2:**
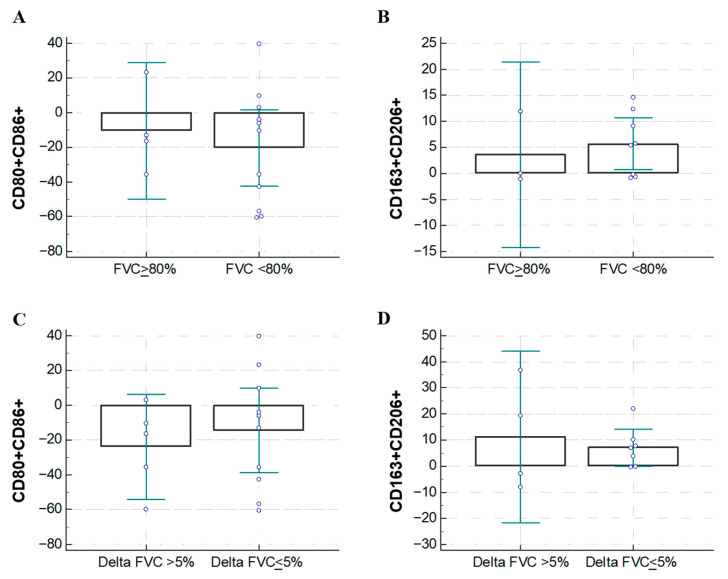
Effect of NTD on macrophage phenotype in relation to the Forced Vital Capacity. (**A**) Variation between T0 and T1 of the expression of CD80CD86 in the M1-like macrophage population: differences between patients who have more or less than 80% of FVC at the basal; (**B**) variation between T0 and T1 of the expression of CD206CD163 in the M2-like macrophage population: differences between patients who have more or less than 80% of FVC at baseline; (**C**) variation between T0 and T1 of the expression of CD80CD86 in the M1-like macrophage population: differences between patients who have more or less than 5% of FVC at the basal; (**D**) variation between T0 and T1 of the expression of CD206CD163 in the M2 macrophage population: differences between patients who have lost more or less than 5% of FVC between T0 and T1. Data were analyzed by t-test and presented as box-and-whisker plots showing the median (center line), interquartile range (box) and minimum–maximum values (whiskers) of the percentage of positive cells on CD14+ population, each circle represents a measure.

Interestingly, patients with a more preserved function (FVC greater than 80%) expressed lower M1 (CD80CD86) markers (Figure 2A) and an increased M2 pro-fibrotic marker pattern (CD206CD163) (Figure 2B), which was non-statistically significant. Moreover, patients with a rapid decline in FVC after 6 months of treatment expressed a non-significant trend of decreased M1 markers (CD80CD86) (Figure 2C) and increased M2 markers (CD206CD163) (Figure 2D) compared to patients with a slower functional decline in FVC.

### 3.3. NTD Increases Oxidative Burst in Macrophages

**Figure 3:**
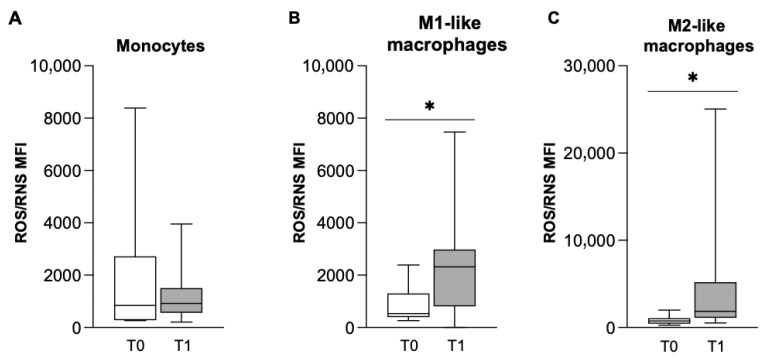
Effect of NTD on oxygen and nitrogen reactive species production in monocytes (**A**), M1-like macrophages (**B**) and M2-like macrophages (**C**) at T0 (white bar) and T1 (gray bar). FACS analysis of ROS/RNS producing cells. Data were analyzed by the Wilcoxon–Mann–Whitney test and presented as box-and-whisker plots showing the median (center line), interquartile range (box) and minimum–maximum values (whiskers) of the mean of the fluorescence intensity (MFI) of 16 different patients. Statistical significance: * *p* < 0.05 versus T0.

In order to explore the activation state of cells, FACS analysis was performed to evaluate the production of a bulk of ROS and RNS (H_2_O_2_, ONOO^−^, HO, NO, ROO and O_2_^−^). As shown in Figure 3A, the median fluorescence intensity (MFI) of monocytes was not affected by 6 months of treatment with NTD, while we observed a significant increase in M1-like and M2-like MDM populations at T1 (Figure 3B,C), a result that confirms the macrophage polarization toward the pro-inflammatory phenotype, as M2-macrophages are generally low ROS producers.

### 3.4. Effect of NTD on Inflammatory Cytokines and Chemokines

**Figure 4:**
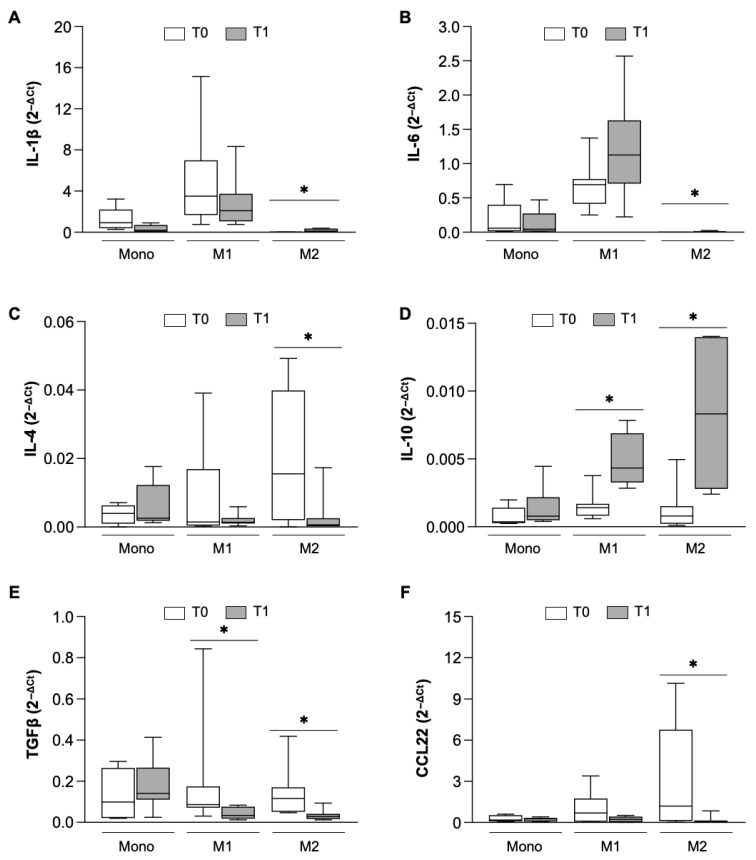
Gene expression analysis of cytokines and chemokines in monocytes (Mono) and M1 and M2 macrophages. Real time analysis of IL-1β (**A**), IL-6 (**B**), IL-4 (**C**), IL-10 (**D**), TGF β (**E**), and CCL22 (**F**) at T0 (white bar) and T1 (gray bar). Data were analyzed by the Wilcoxon–Mann–Whitney test between T0 and T1, expressed as 2^−DCt^ and presented as box-and-whisker plots showing the median (center line), interquartile range (box) and minimum–maximum values (whiskers) of 8 different patients. Statistical significance: * *p* < 0.05 versus T0.

**Figure 5:**
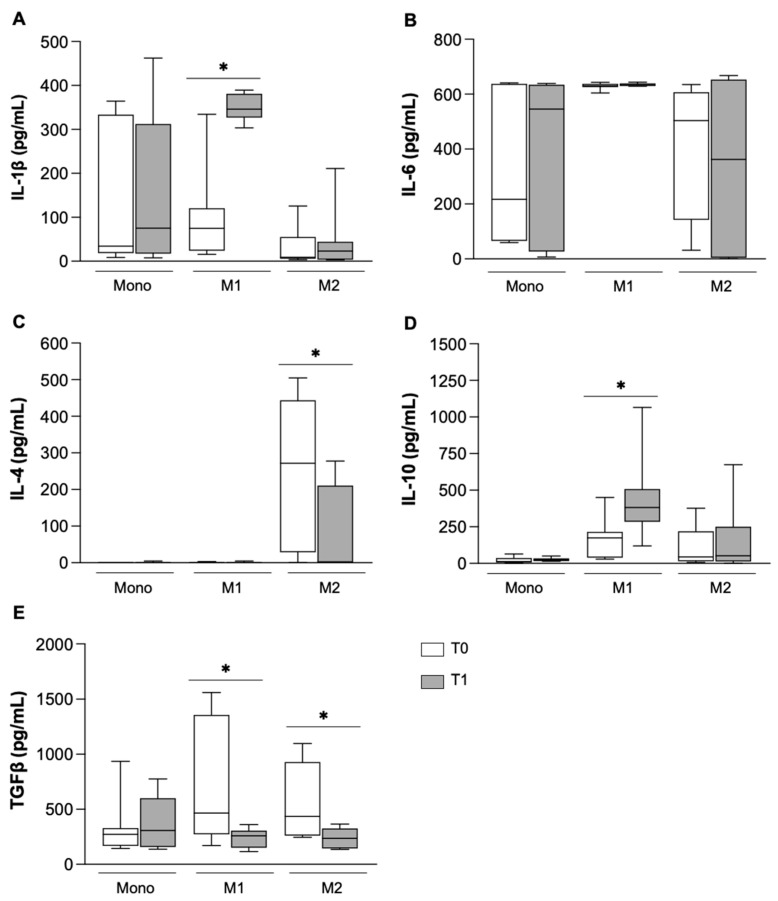
Cytokine release in monocytes (Mono) and M1-like and M2-like macrophages. ELISA analysis of IL-1β (**A**), IL-6 (**B**), IL-4 (**C**), IL-10 (**D**) and TNFα (**E**) at T0 (white bar) and T1 (gray). Data were analyzed by the Wilcoxon–Mann–Whitney test between T0 and T1, expressed as pg/mL and presented as box-and-whisker plots showing the median (center line), interquartile range (box) and minimum–maximum values (whiskers) of at least 6 different patients. Statistical significance: * *p* < 0.05 versus T0.

To better define the immunomodulatory effect of NTD, the expression (Figure 4) and release (Figure 5) of some specific cytokines was further evaluated in monocytes and MDM from IPF patients after 6 months of treatment. In line with the oxidative stress and phenotype results, no differences were observed in the gene expression of any of the analyzed cytokines in monocytes isolated before and after treatment with NTD (Figure 4A–F).

On the contrary, NTD significantly affected the mRNA levels of IL-1β, IL-6, IL-4, IL-10, TGFb and CCL22 in M1-like and/or M2-like macrophages. In fact, the expression of IL-1β (Figure 4A), IL-6 (Figure 4B) and IL-10 (Figure 4D) was significantly increased in M2 macrophages, while the expression of IL-4 (Figure 4C), TGFβ (Figure 4E) and CCL22 (Figure 4F) was significantly reduced, indicating that cells in the M2-like population were triggered to shift toward an anti-fibrotic phenotype. In the M1-like macrophage population, the expression of IL-10 was enhanced (Figure 4D) and the TGFβ was notably decreased (Figure 4F), sustaining the anti-fibrotic potential of these cells. We then investigated whether cytokine release was also affected by NTD (Figure 5).

While monocyte activity was not modulated, confirming previously reported results (Figure 5A–E), data obtained in macrophages confirmed the immunomodulatory ability of NTD on them. In fact, NTD supported the anti-fibrotic activity of M1-like MDM, triggering a significant release of IL-1β (Figure 5A) and IL-10 (Figure 5D), and reducing the release of IL-4 (Figure 5C) in M2-like macrophages and of TGFβ (Figure 5E) both from M1-like and M2-like MDM.

## 4. Discussion

The manipulation of macrophage polarization by an anti-fibrotic drug can be instrumental in slowing the progression of interstitial lung disease such as IPF. In fact, the imbalance between the two main macrophage subtypes could favor or contrast aberrant deposition of the matrix in pulmonary parenchyma. Roughly, M1 macrophages are considered anti-fibrotic, while M2 are pro-fibrotic, and the macrophage orchestrating lung homeostasis can have different origins. In particular, it is possible to distinguish interstitial macrophages (IM) and alveolar macrophages (AM). The latter are the most abundant resident immune cells in lungs and derive from circulating monocytes that are recruited by the onset of IPF to replenish the pool of alveolar macrophages [37]. AM-derived monocytes have been demonstrated to be mainly alternative macrophages, the major producers of TGF-β, which consequently promotes matrix deposition and thus fibrosis progression [38]. Hence, blocking monocyte recruitment in the fibrotic niche of IPF-injured lungs attenuates the fibrotic process [38,39,40]. In fact, it has been demonstrated in an animal model that the phenotype of the AM-derived monocytes reflects the state of the precursor circulating monocytes and not only the lung environment [41]. In this context, we carried out experiments to evaluate the possible immunomodulatory effect of NTD in monocytes isolated from IPF patients under treatment that could then be reflected on the macrophage populations differentiated from them. Unfortunately, the number of enrolled patients is limited, and since we were unable to obtain lung tissue biopsies or bronchoalveolar lavage (BAL) to isolate AM, we restricted the analyses to circulating monocytes, which are known to be recalled to the lung during an inflammatory process to increase the macrophage pool [37]. We have demonstrated that the anti-fibrotic drug NTD is able to prime circulating monocytes to differentiate mainly toward M1 macrophages despite the environmental stimuli. In fact, although no differences regarding phenotype and activity were observed between circulating monocytes isolated from IPF patients before (T0) and after 6 months (T1) of NTD treatment, the predisposition of NTD-monocytes at T1 to acquire an anti-fibrotic phenotype was demonstrated by the significant increase in CD80+CD86+ cells, even in the presence of the experimental cytokine cocktail (IL-4, IL-10, IL-13) favorable for an M2-like population. These results are in line with a recent study of Soldano et al. [19], that firstly reported NTD’s in vitro capability to downregulate the pro-fibrotic M2 macrophage phenotype [19], reducing the expression of CD204, CD206 and CD163 [1]. These surface markers are representative for activated M2 macrophages and are involved in the orchestration of the fibrotic state, as CD163 is a scavenger receptor for the hemoglobin/haptoglobin complex and modified LDL, as well as binding several bacterial ligands [42], while CD206 is the mannose receptor known to play a role in tissue homeostasis and regeneration [43,44]. Moreover, Nouno et al. [45] have demonstrated that the overexpression of these surface markers is representative of a severe clinical outcome in IPF patients. With our study, we demonstrate that NTD is able to predispose circulating monocytes isolated from NTD-treated patients to acquire a pro-inflammatory phenotype, since their MDMs, despite being cultured with M2-induction medium, release low levels of TGFβ, CCL22 and IL-4, but at the same time, high levels of IL-10. In particular, TGFβ is a master regulator of fibrosis [46,47], mainly stimulating the secretome of fibroblasts and activating other cell types, such as epithelial and vascular cells inducing a pro-fibrotic phenotype [48]. Moreover, in IPF-injured tissues, leukocytes, platelets and macrophages are recruited, leading to the secretion of pro-fibrotic cytokines (e.g., TGFb) and consequently to chronic fibroblast activation, proliferation and matrix deposition [49]. Macrophages are supposed to be the major source of TGFβ, since it has been reported that the inhibition of monocyte/macrophage recruitment in the injured tissues substantially reduces this cytokine secretion, and consequently fibrosis [50,51]. However, fibroblasts are also recognized to play an important role in the initiation of the fibrotic process [52], with dynamic crosstalk between macrophages and fibroblasts that mutually self-maintain the fibrotic process by releasing inflammatory and profibrotic cytokines, including TGFβ and IL-6 [53,54]. In M1-like and M2-like macrophage populations derived from monocytes from NTD-treated patients, TGFβ was strongly reduced, both in terms of gene expression and protein secretion. This downregulation was sustained by the reduction in CCL22 and IL-4, whose release exacerbates the fibrotic process [55,56], and the increase in IL-10 secretion that has been recently reported to suppress the TGFβ-dependent fibrosis both in vitro and in vivo [57,58]. The overall analysis of the cytokine release modulation of macrophages induced by NTD treatment leads to a reasonable speculation about a drug-dependent development of an immunomodulatory microenvironment that counteracts matrix deposition by fibroblasts, effectively interrupting the mutual influence on each other’s behavior [59]. How NTD affects IL-10 expression and release is quite elusive. IL-10 is recognized as having a dual role, acting as both an anti-inflammatory and anti-fibrotic cytokine; therefore, its elevated levels in IPF patients is still a controversial issue, as it is not yet clear whether it is a disease-triggering event by acting as a pro-fibrotic cytokine [60,61], or whether it is a protective event by acting as an anti-inflammatory and anti-fibrotic cytokine [62,63]. The mechanism underlying the increase in IL-10 by NTD has not been investigated by us, but our results support the hypothesis that NTD may contribute to the reduction in the fibrotic process, thanks to immunomodulation at the macrophage level with a reduction in TGF-β and increase in IL-10 levels by exploiting its protective role. Our ex vivo results on MDM could be instrumental to understanding the immunomodulatory role of NTD. Indeed, during IPF onset and progression, the prevalence of M1 or M2 macrophages undergoes to subsequent changes favoring or counteracting the disease development [64]. As summarized by Ge et al. [64], macrophages in IPF have varying roles throughout disease progression, with M1 macrophages being pro-inflammatory in early stages, while M2 macrophages exhibit anti-inflammatory and fibrotic effects in later stages. Imbalance between M1 and M2 macrophages may drive IPF progression [26]. In our case series, we found no statistically significant findings when considering the FVC data. However, when considering the DLCO data, we found a reduction in CD80+CD86+ markers in M2 macrophages, and an increase in CD163+CD206+ in the population with the lowest DLCO. It would therefore appear that in this group of patients, with the limitation that the DLCO does not fully reflect the progression of the disease but is still an indicator of interstitial damage, pro-fibrotic markers are more expressed and pro-inflammatory are less, as if to define a less inflammatory and more fibrotic state of the disease. Moreover, the change from T0 to T1 of the M2-like macrophage markers in patients with FVC < 80 is indicative of the immunomodulatory effect of the NTD. In this context, NTD treatment could promote the establishment of an anti-fibrotic environment, resulting in a prevalence of M1 macrophages. Furthermore, we could speculate that NTD contributes to the initial establishment of anti-fibrotic status that is, in turn, sustained by the secretome of primed immune cells. Although the data are not statistically significant, from a translational point of view, it is possible to hypothesize that, in IPF patients treated with NTD, the monocytes recruited to the injured lung are more prone to generating an anti-fibrotic macrophage population. Since the Mo-AMs persist on the lung for years after recruitment [38], we can speculate that NTD immunomodulation of monocytes/macrophages could help to slow the fibrotic process, ameliorating the patient prognosis.

## 5. Study Limitation

Our findings should be interpreted despite some limitations of the study, as mentioned before. First, the relatively low number of enrolled patients is certainly responsible for the lack of significance of some tests. Nonetheless, the data still represent an important indication of the effects of NTD on monocyte/macrophage phenotype and responsiveness. Second, although the protocols used for the isolation of monocytes and for their differentiation into the two macrophage subtypes are approved and accepted by the scientific community, we certainly cannot ascertain that the cell populations obtained recreate the physiological/pathological milieu 100%, but they are nevertheless indicative. Furthermore, a direct analysis of whole blood or PBMCs would certainly have provided a more direct indication of nintedanib’s action on cells in vivo, which could be influenced by the manipulation of monocyte isolation and differentiation. Third, the BAL would certainly have helped to further investigate the effects of the NTD, not only on the alveolar macrophages but also on monocytes, whose numbers are low in healthy individuals but significantly increase in patients with IPF. Unfortunately, while at T0, the BAL is considered diagnostic and therefore appropriate, at T1, it is considered unethical to subject patients to BAL solely for research purposes. Since we could not evaluate the changes induced by NTD from T0 to T1, we considered it useless to analyze the BAL only at the basal state.

## 6. Conclusions

Our results show that although it does not have a direct effect on monocytes, NTD drives the polarization of monocyte-derived macrophages toward the classical M1-like anti-fibrotic phenotype, highlighting an immunomodulatory effect of NTD. This data leads us to hypothesize that NTD could induce this shift even in contributing to an improvement in the patient’s fibrotic state.

## Figures and Tables

**Table 1:** Baseline characteristics of the enrolled IPF patients.

Age, in years (mean ± SD)	73.3 ± 6.3
BMI (mean ± SD)	24.7 ± 3.8
Smoking history (former, non-smoker)	22%
UIP-CT	
Definite	9/20–45%
Probable	11/20–55%
Patients in treatment with prednisone (max dose of admitted oral prednisone 5 mg/day)	6/20–30%
T0	
FVC % of the predicted value (mean ± SD)	70.8 ± 13.1%
TLC % of the predicted value (mean ± SD)	63.3 ± 18.5%
DLCO % of the predicted value (mean ± SD)	52.5 ± 12.7%
Monocyte count, in cells/µL (mean ± SD)	0.55 ± 0.2 cells/µL

FVC = Forced Vital Capacity; TLC = Total Lung Capacity; DLCO = Diffusing Capacity of Lung Carbon Monoxide; and UIP-CT = Usual Interstitial Pneumonia Pattern on Computer Tomography.

## Data Availability

Data available upon request due to restrictions (privacy).

## Supplementary Materials

The following supporting information can be downloaded at: https://www.mdpi.com/article/10.3390/biom16020319/s1, Table S1: Changes of pathological characteristics of IPF patients at T1; Table S2: Monocytes and macrophages of patients stratified basing on Usual Interstitial Pneumonia Pattern at computer tomography (UIP-CT) evaluation: probable vs definite; Table S3: Monocytes and macrophages of patients stratified basing on Diffusing Capacity of Lung Carbon Monoxide (DLCO) values: >60%, 40-60% and <40%; Table S4: Monocytes and macrophages of patients stratified basing on monocytes count: <60% and >60%; Table S5: Monocytes and macrophages of patients stratified basing on FVC: <80% and > 80%; Figure S1: Gating strategy for FACS analysis of monocytes; Figure S2: Gating strategy for FACS analysis of MDM.
