# Supplementary material for: Immunomodulatory Effects of Nintedanib on Human Blood Monocytes/Macrophages from Patients with Idiopathic Pulmonary Fibrosis
Source: Biomolecules. 2026 Feb 18;16(2):319. doi: 10.3390/biom16020319 (PMC12938255; doi:10.3390/biom16020319)
Supplement: Supplementary file 1 [file biomolecules-16-00319-s001.zip › biomolecules-4123849-supplementary.pdf]

biomolecules

MDPI

Article

Immunomodulatory effects of nintedanib on human blood monocytes/macrophages from patients with idiopathic pulmonary fibrosis

Supplementary Material

1. Supplementary Tables

**T1**	
FVC % of the predicted value (mean ±SD)	68.1 ±14.9%
TLC % of the predicted value (mean ±SD)	57.2 ±15.8%
DLCO % of the predicted value (mean ±SD)	42.2 ±13.8%

Supplementary table S1. Changes of pathological characteristics of IPF patients at T1. FVC = Forced Vital Capacity; TLC = Total Lung Capacity; DLCO = Diffusing Capacity of Lung Carbon Monoxide.

A)

		**Probable (% mean±SEM)**	**Definte (% mean±SEM)**
		**n=11**	**n=9**
		**Monocytes**	
	**CD14+CD16+**	11 ± 2	20 ± 5
	**CD14++CD16+**	7 ± 3	**3 ± 1 (*)**
	**CD14++CD16-**	34 ± 5	**17 ± 4 (*)**
**UIP-CT**		**M1-like macrophages**	
	**CD80+**	22 ± 3	27 ± 7
	**CD86+**	65 ± 9	53 ± 10
	**CD80+CD86+**	14 ± 2	24 ± 5
	**CD163+**	26 ± 7	18 ± 4
	**CD206+**	15 ± 2	14 ± 3
	**CD163+CD206+**	8 ± 2	3 ± 1
		**M2-like macrophages**	
	**CD80+**	15 ± 4	17 ± 4

	**CD86+**	61 ± 9	44 ± 10
	**CD80+CD86+**	11 ± 3	12 ± 3
	**CD163+**	33 ± 8	23 ± 5
	**CD206+**	17 ± 3	14 ± 4
	**CD163+CD206+**	11 ± 4	5 ± 3

B)

		**Probable (mean±SEM)**	**Definte (mean±SEM)**
		**n=3**	**n=17**
		**Monocytes**	
**UIP-CT**	nmol CytC red/10^6 cells	2 ± 1	**6 ± 2 (*)**
		**M1-like macrophages**	
	nmol CytC red/10^6 cells	6 ± 1	7 ± 2
		**M2-like macrophages**	
	nmol CytC red/10^6 cells	1 ± 0.2	**5 ± 2 (*)**

Supplementary Table S2. Monocytes and macrophages of patients stratified basing on Usual Interstitial Pneumonia Pattern at computer tomography (UIP-CT) evaluation: probable vs definite. A) Phenotypic characterization by cytofluorimetric analysis. *$p<0.05$ vs probable group. B) Indirect analysis of basal superoxide anion production by resting cells. Data are expressed as mean ± SEM of nmoles of cytochrome C reduced/106 cells. *$p<0.05$ vs probable group.

A)

		>60 (% mean±SEM)	40-60 (% mean±SEM)	<40 (% mean±SEM)
		**n=3**	**n=14**	**n=3**
		**Monocytes**		
**DLCO**	**CD14+CD16+**	14 ± 9	17 ± 3	11 ± 4
	**CD14++CD16+**	3 ± 0.3	6 ± 2	2 ± 1
	**CD14++CD16-**	37 ± 14	26 ±4	17 ± 7
		**M1-like macrophages**		
	**CD80+**	30 ± 9	33 ± 6	27 ± 12
	**CD86+**	81 ± 11	70 ± 7	51 ± 14
	**CD80+CD86+**	24 ± 6	22 ± 6	21 ± 13
	**CD163+**	40 ± 7	27 ± 6	7 ± 6
	**CD206+**	5 ± 3	15 ± 2	17 ± 8
	**CD163+CD206+**	4 ± 2	7 ± 2	5 ± 5
		**M2-like macrophages**		
	**CD80+**	28 ± 8	16 ± 3	15 ± 7
	**CD86+**	**81 ± 5 (*)**	**59 ± 7 (*)**	**16 ± 8**
	**CD80+CD86+**	15 ± 8	11 ± 2	8 ± 6
	**CD163+**	59 ± 12	28 ± 6	23 ± 13
	**CD206+**	12 ± 7	15 ± 3	25 ± 13
	**CD163+CD206+**	10 ± 6	8 ± 3	12 ± 11

B)

		>60 (mean±SEM)	40-60 (mean±SEM)	<40 (mean±SEM)
		**n=3**	**n=14**	**n=3**
		**Monocytes**		
**DLCO**	**nmol CytC red/10^6 cells**	6 ± 2	3 ± 1	12 ± 6
		**M1-like macrophages**		
	**nmol CytC red/10^6 cells**	6 ± 2	7 ± 1	5 ± 1
		**M2-like macrophages**		
	**nmol CytC red/10^6 cells**	4 ± 4	2 ± 1	6 ± 3

Supplementary Table S3. Monocytes and macrophages of patients stratified basing on Diffusing Capacity of Lung Carbon Monoxide (DLCO) values: >60%, 40-60% and <40%. A) Phenotypic characterization by cytofluorimetric analysis. Data are expressed as mean ± SEM of percentage of positive cells. *p<0.05 vs <40 group. B) Indirect analysis of basal superoxide anion production by resting cells. Data are expressed as mean ± SEM of nmoles of cytochrome C reduced/10^6 cells.

A)

		**<60 (% men±SEM)**	**>60 (% men±SEM)**
		***n*=17**	***n*=6**
		**Monocytes**	
**Monocytes count**	**CD14+CD16+**	15 ± 2	18 ± 7
	**CD14++CD16+**	4 ± 1	3 ± 1
	**CD14++CD16-**	23 ± 4	30 ± 8
		**M1-like macrophages**	
	**CD80+**	27 ± 4	26 ± 8
	**CD86+**	61 ± 8	65 ± 10
	**CD80+CD86+**	18 ± 3	22 ± 7
	**CD163+**	21 ± 5	26 ± 11
	**CD206+**	12 ± 2	21 ± 3
	**CD163+CD206+**	6 ± 2	7 ± 3
		**M2-like macrophages**	
	**CD80+**	16 ± 3	15 ± 4
	**CD86+**	51 ± 8	51 ± 14
	**CD80+CD86+**	12 ± 2	10 ± 3
	**CD163+**	27 ± 6	31 ± 8
	**CD206+**	15 ± 3	18 ± 6
	**CD163+CD206+**	9 ± 3	9 ± 5

B)

		**<60 (men±SEM)**	**>60 (men±SEM)**
		n=17	n=6
		**Monocytes**	
**Monocytes count**	**nmol CytC red/10^6 cells**	3 ± 1	3 ± 1
		**M1-like macrophages**	
	**nmol CytC red/10^6 cells**	6 ± 1	7 ± 2
		**M2-like macrophages**	
	**nmol CytC red/10^6 cells**	3 ± 1	2 ± 2

Supplementary Table S4. Monocytes and macrophages of patients stratified basing on monocytes count: <60% and >60%. A) Phenotypic characterization by cytofluorimetric analysis. Data are expressed as mean ± SEM of percentage of positive cells. B) Indirect analysis of superoxide anion produced basically by cells. Data are expressed as mean ± SEM of nmoles of cytochrome C reduced/10^6cells.

A)

		<80 (% men±SEM)	>80 (% men±SEM)
		**n=17**	**n=6**
		**Monocytes**	
	**CD14+CD16+**	12 ± 2	25 ± 6
	**CD14++CD16+**	6 ± 2	3 ± 1
	**CD14++CD16-**	25 ± 5	23 ± 6
		**M1-like macrophages**	
	**CD80+**	23 ± 4	28 ± 7
	**CD86+**	57 ± 8	72 ± 7
**FVC**	**CD80+CD86+**	18 ± 3	21 ± 5
	**CD163+**	19 ± 5	30 ± 8
	**CD206+**	14 ± 2	18 ± 4
	**CD163+CD206+**	6 ± 2	6 ± 3
		**M2-like macrophages**	
	**CD80+**	15 ± 3	20 ± 6
	**CD86+**	45 ± 8	66 ± 10
	**CD80+CD86+**	10 ± 2	14 ± 4
	**CD163+**	25 ± 5	34 ± 12
	**CD206+**	15 ± 3	18 ± 6
	**CD163+CD206+**	8 ± 2	11 ± 8

B)

		<80 (% men±SEM)	>80 (% men±SEM)
		***n*=17**	***n*=6**
		**Monocytes**	
	**nmol CytC red/10^6 cells**	3 ± 1	3 ± 1
**FVC**		**M1-like macrophages**	
	**nmol CytC red/10^6 cells**	7 ± 1	6 ± 2
		**M2-like macrophages**	
	**nmol CytC red/10^6 cells**	1 ± 0.5	5 ± 3

Supplementary Table S5. Monocytes and macrophages of patients stratified basing on FVC: <80% and >80%. A) Phenotypic characterization by cytofluorimetric analysis. Data are expressed as mean ± SEM of percentage of positive cells. B) Indirect analysis of cell superoxide anion basal production. Data are expressed as mean ± SEM of nmoles of reduced cytochrome C /10^6 cells.

2. Supplementary Figures

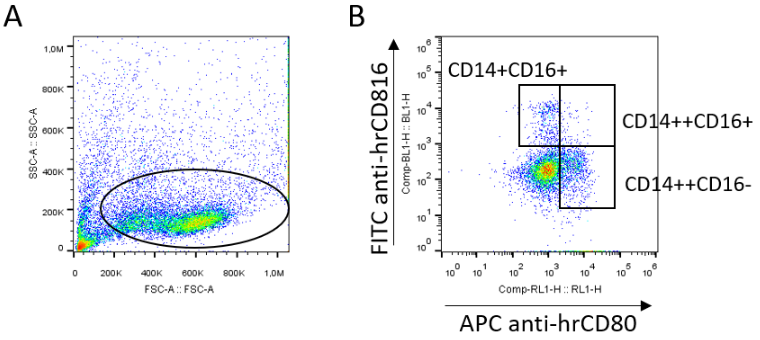

Supplementary Figure S1. Gating strategy for FACS analysis of monocytes. A) FSC-SSC dot plot. B) Monocyte gates on CD14-CD16 dot plot.

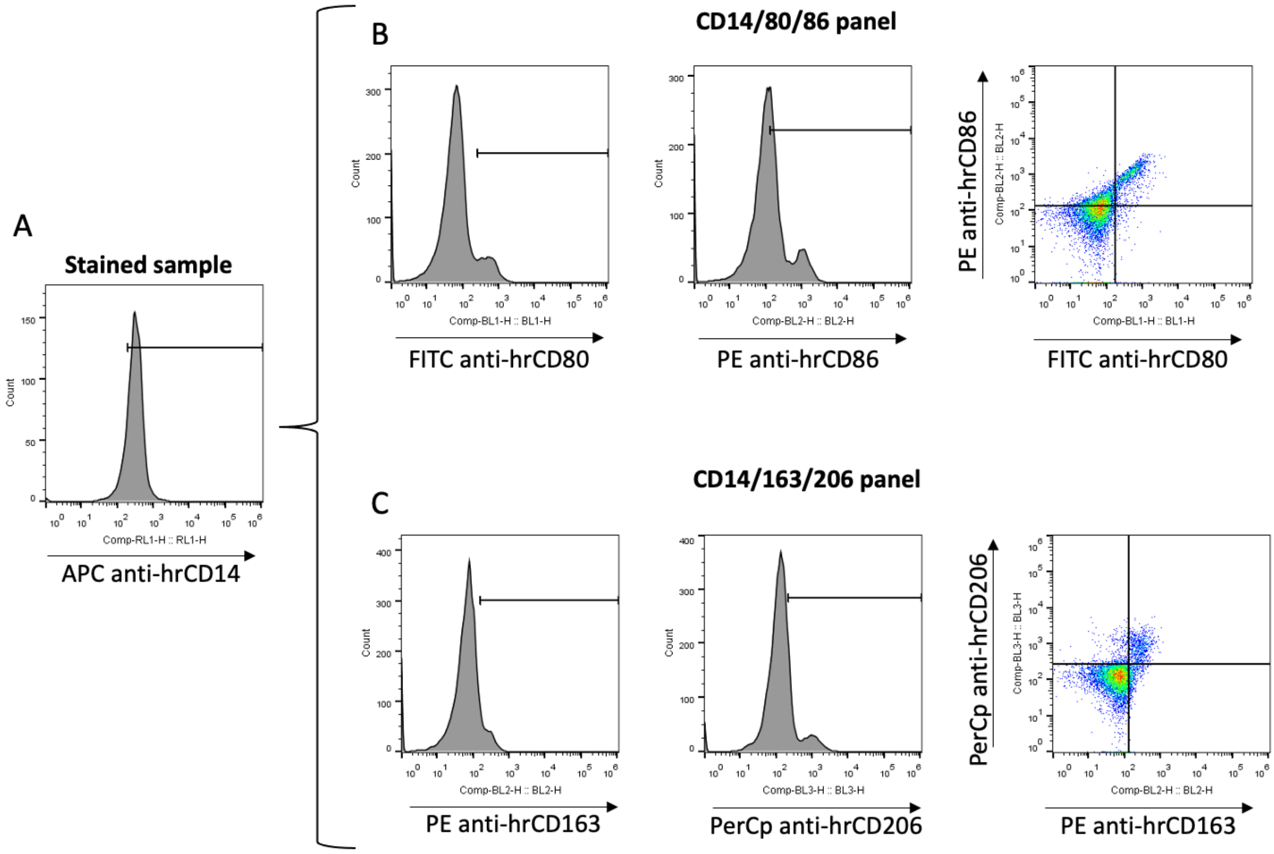

Supplementary Figure S2. Gating strategy for FACS analysis of MDM. A) Gate set on CD14 histogram B) Histograms and dot plot to analyze CD14/CD80/CD86 marked cells. C) Histograms and dot plot to analyze CD14/CD163/CD206 marked cells.
